# Robot-assisted assessment of muscle strength

**DOI:** 10.1186/s12984-017-0314-2

**Published:** 2017-10-11

**Authors:** Marco Toigo, Martin Flück, Robert Riener, Verena Klamroth-Marganska

**Affiliations:** 1 Laboratory for Muscle Plasticity, Balgrist University Hospital, University of Zurich, Zurich, Switzerland; 2 Sensory-Motor Systems Lab, Department of Health Sciences and Technology ETH Zurich, Zurich, Switzerland; 3 Balgrist University Hospital, University of Zurich, Zurich, Switzerland

**Keywords:** Neuromuscular, Upper extremity, Robot, Assessment, Neurorehabilitation, Sensorimotor

## Abstract

Impairment of neuromuscular function in neurological disorders leads to reductions in muscle force, which may lower quality of life. Rehabilitation robots that are equipped with sensors are able to quantify the extent of muscle force impairment and to monitor a patient during the process of neurorehabilitation with sensitive and objective assessment methods. In this article, we provide an overview of fundamental aspects of muscle function and how the corresponding variables can be quantified by means of meaningful robotic assessments that are primarily oriented towards upper limb neurorehabilitation. We discuss new concepts for the assessment of muscle function, and present an overview of the currently available systems for upper limb measurements. These considerations culminate in practical recommendations and caveats for the rational quantification of force magnitude, force direction, moment of a force, impulse, critical force (neuromuscular fatigue threshold) and state and trait levels of fatigue.

## Background

*This work was developed in the frame of the project “State of the Art Robot-Supported assessments (STARS)” as part of the COST Action TD1006 “European Network on Robotics for NeuroRehabilitation”* [1]*. STARS is intended to equally serve clinical practitioners and scientists working in the field of neurorehabilitation. The goal is to give recommendations for development, implementation, and administration of different indices of robotic assessments, grounded on the scientific literature available at this time.*

Intact neuromuscular function is indispensable for motor function, activities of daily living and social participation [2]. Neurological disorders can result in severe impairment of neuromuscular function. In stroke, the muscular weakness results from changes in muscle mass, length, muscle architecture (e.g., pennation angle) muscle composition (i.e., fiber type, fat content, connective tissue) and material properties [3]. Furthermore, an increase in stretch reflex excitability, antagonist muscle coactivation, and a decrease in motor unit firing rate are observed [4]. Sufficient force in the upper limb is related to the ability to adequately perform many activities of daily living [2], and regaining muscle force is a major goal in upper extremity neurorehabilitation. Furthermore, grip strength is a major predictor of recovery and all-cause mortality [5, 6]. To provide optimal therapy, valid, reliable, sensitive, and standardized assessment methods are crucial as they serve to quantify the extent of impairment, to identify the most effective and time efficient training and to progressively adapt the therapy (exercise type, intensity and time commitment) to the individual’s progress, needs and goals.

Force assessments with either quantitative or more qualitative methods is an integral part of the physical examination in neurorehabilitation such as stroke [7] or spinal cord injury (SCI, [8]). The manual muscle test (MMT) is the most frequently used clinical assessment. It is integrated in the international standards for neurological classification of spinal cord injury [8]. It is classified as a semi-quantitative method with relatively low accuracy and sensitivity. Isometric force of individual muscles and muscle groups is subjectively rated based on the effective performance of a movement against gravity or resistance applied by an examiner. A number of grading systems exist for manual muscle testing [9–11]. Quantification of force or moment with continuous variables requires instrumentation. A common method in clinical practice is isometric dynamometry with a handheld dynamometer [12]. It has good reliability and it is sensitive for all grades. Furthermore, it is less dependent on technique than the MMT, but it is not suitable when movement against resistance cannot be performed. Isometric dynamometry can be integrated in clinical tests (e.g., the Wolf Motor Function Test [13]).

Novel and rapidly expanding technologies such as rehabilitation robotics can provide objective quantification of neuromuscular function in a standardized way. This may help to overcome the common limitations of clinical assessments [14], and advance the development of personalized human-machine interfaces alongside assessments and rehabilitation interventions that are tailored to a specific patient’s anatomy and neurological disorder. Attempts have been made to correlate robotic mechanical variables with established clinical scores based on the International Classification of Functioning, Disability and Health (ICF, http://www.who.int/classifications/icf/en/) [14]. In this article, we will present recommendations for the design and application of robotic assessments of muscle force in the field of upper limb neurorehabilitation. The major aim of this paper is not to present rigorous mathematical descriptions of already existing assessment methods but rather to identify the relevant practical and theoretical concepts of muscle physiology that should underlie all robotic assessments of muscle function and to highlight the various manifestations of muscle force exertion that need to be quantified. We will restrict our work on electromechanical and robot-assisted devices; additional means such as electromyogram (EMG) are discussed in the context of neuromechanical interplay.

## Review

### Definition of measurements of strength

#### The function of skeletal muscle and its neural control

The colloquial term “strength” usually refers to the SI-entities mass, force, moment or power. In the context of human movement, the function of muscle is to exert force and it does so by acting either exclusively or in combination on shortening or lengthening the muscle-tendon unit or by keeping its length quasi-constant. For the purpose of this review we will refer to these three modes of force exertion as being miometric, pliometric, and isometric, respectively [15]. The neural control strategies underlying these modes of muscle action can differ. Whereas the recruitment order of motor units is similar during submaximal miometric and pliometric actions and consistent with the size principle of motor unit recruitment, the discharge rate is systematically lower during pliometric actions as compared to miometric actions [16–19]. Furthermore, untrained individuals are usually unable to fully activate their muscles despite a maximal intended pliometric action [20, 21]. In order to pinpoint muscle function deficits to neural impairments, it might be valuable to assess muscle function for the three types of force exertion modes separately and in combination.

### Robotic systems for strength measurement

#### Isokinetic dynamometers

The clinical gold standard for the assessment of musculoskeletal performance is the isokinetic (isokinetic for “same motion”) dynamometer (ID). IDs are devices that measure joint moment while maintaining a constant joint velocity or rather a constant angular velocity of the machine’s lever against which muscle action occurs. The ID movements are two-dimensional and rotational around a joint. The way for creating moment may be hydraulic, frictional or electro-magnetic and the angle-moment curves between these systems may differ considerably [22]. Most devices allow also for isometric measurements.

Although ID measurements are sensitive, reproducible, and can contribute valuable information, there are a number of caveats that concern the validity of interpretations from “isokinetic” assessment results:

1. In spite of the fact that angular velocity is kept constant during isokinetic assessments, muscle fiber velocity is not constant throughout the range of motion (ROM), especially at higher velocities [23]. This is due to various factors, such as variable muscle moment arm, variable tendon compliance, and variable muscle activation throughout the ROM.
2. The term “isokinetic” refers to the angular velocity of the device and not to muscle fiber or limb velocity [24], and, although IDs control for velocity, phases of accelerations, oscillations and decelerations limit the periods of constant angular velocity [25].
3. Angular velocity of many “normal” activities is clearly beyond the maximal angular velocity (~5.24 rad/s, i.e. ~ 300°/s) of typical isokinetic devices. For instance, humeral internal rotation velocity during throwing (overhand pitching) may reach 130–134 rad/s and knee and ankle angular velocity during jumping is about 12–15 rad/s [26–28].
4. The typically employed miometric actions on an ID cannot a priori yield maximal force values [29]. Pliometric actions on an ID cannot yield maximal moment values either, because the moment record itself is often of shorter duration than the time required to accomplish complete muscle fiber activation. Even for isometric actions it takes at least 400–700 ms up to several seconds to reach peak moment [30, 31].
5. Movement in an ID requires both spatial and temporal coordination. The movement direction in IDs defines only the path of the attached part of the limb (e.g., the hand). Although being planar, several muscles and muscle compartments have to be recruited by neurons in a coordinated, timed manner. Thus, a low magnitude in the resulting force (especially during fast movements) could be misinterpreted as muscle weakness, when indeed the low force is caused by suboptimal force directions and decreased temporospatial coordination due to neuronal deficits. It has to be emphasized that muscle architecture changes with neurological deficits and the recruitment required for a defined movement may differ in patients from that of healthy subjects. Six-axes sensors can detect forces from all three Cartesian coordinates (x, y and z) and recognize deviations of the force direction. The information may also be used for smoothness assessments.
6. The moments obtained using IDs are not the same as the resultant joint moments. Gravitational moments are a major source of error and must be corrected for. They are usually modeled using sine or cosine functions and can be corrected by either subtracting (i.e., when working with gravity) or adding (i.e., when working against gravity) the limb weight [32]. Furthermore, passive elastic moments contribute to the total joint moment. The term describes the passive viscoelastic deformation primarily by tissues crossing the joint, including the muscles, the tendons and ligaments [32]. In the shoulder, it can be used to store and release energy (e.g., during throwing [33]).
7. IDs measure the moment at their own axis and do not account for the variation of the rotational axis of a joint or misalignment of the limb with the lever arm, which may additionally change during movement inertia.

#### Endeffector and exoskeleton robots for force assessments

Therapy robots are often equipped with force sensors for quantifying interaction forces between the device and the user. The devices provide raw sensor data about force during functional movements and these data can be used to extract complex pathology features such as abnormal synergies [34]. Just as for the dynamometers, misalignment with a device and variation of the rotational axis of a joint can distort results. Furthermore, the subject has to interact with the dynamics of the device, which includes joint friction, mass and inertia, as well as backlash. Even with an online adapting compensation, a robot will not achieve full transparency. This may confound the results and even hamper assessments in very weak patients. Several approaches in both endeffector and exoskeleton robots were assessed in healthy participants and patients:

#### MIME

The Mirror Image Movement Enabler (MIME) is a 6 degrees of freedom (DOF) endeffector robotic device for shoulder and elbow neurorehabilitation. It is equipped with a 6-axis force sensor that allows for unilateral and bilateral training [35]. Modeled forearm trajectories were used to control point to-point reaching movements in the horizontal plane. The force direction error (FDE, see “Force direction error”) was calculated during passive and active-assisted movements. Furthermore, positive work, potential work (defined as the work that would have been done if the force magnitude was directed precisely in the movement direction and the torque magnitude was oriented precisely in the direction of rotation) and work efficiency (the work done divided by the potential work, had the total force been oriented toward the target at all points along the path) were calculated.

#### ARM guide

The ARM Guide is an endeffector, linear robotic trainer that can be oriented vertically on an elevation axis and horizontally on a yaw axis across an individual’s workspace. An arm splint slides along a linear track with a 6-axis load cell measuring the forces and moments at the interface between the patient and the device. It was used to quantify pathological synergies in the arm [34]. To do so, the “horizontal constraint force”, i.e., the force perpendicular to both the direction of movement and to the gravity vector during reaching and retrieving task was measured in hemiplegic patients [34]. Movements were performed at peak speeds of 0.2 m/s and 0.8 m/s and at two movement amplitudes (full ROM and 0.45 m).

#### BONES

The Biomimetic Orthosis for Neurorehabilitation of the Elbow and Shoulder (BONES) is a 4 DOF, pneumatically-actuated exoskeleton robot for arm neurorehabilitation [36]. In a “coordinated strength measurement” with BONES, patients post-stroke had to perform maximal intended miometric muscle actions at the shoulder (abduction/adduction, flexion/extension, internal/external rotation), elbow (flexion/extension), forearm (pronation/supination), and wrist (flexion/extension). The joint moment production in the desired as well as undesired directions was shown as online visual feedback to the participant by means of the length of a bar. Deviation tolerance was set within a window that represented ±5° of the desired joint position. The highest uncompensated joint torque of 3 trials of each muscle action direction was retained. A single score summarizing all 12 moment outcome measures was then obtained through principle component analysis (PCA) [37, 38].

#### ARMin

ARMin is a 7 DOF exoskeleton robot for neurorehabilitation therapy of the arm. The device was used for isometric moment assessment of the arm. The maximally detectable joint moments of the motors were 59 N m (elbow, shoulder rotation and horizontal movement) to 82 N m (arm elevation). ARMin moved the patient’s arm in fixed predefined positions and held the position*.* Individuals were asked to apply maximal moment in the joints being measured (arm elevation/retroversion, arm abduction/adduction in the horizontal plane, elbow flexion/extension*)* for 5 s. The measured joint moments were derived from the motor current needed to hold the patient’s arm in the defined position. A moving average filter was applied to reduce the effect of single moment peaks [39–41].

### What to measure

In the following paragraphs we will discuss several basic parameters that need to be specified, standardized, and controlled during robotic force assessments to enable scientific data acquisition, analysis, reporting, and comparison. To acquire the parameters, devices should be equipped with sensors that measure time, position and forces or moments.

#### Peak force

Under normal circumstances, internal muscle force cannot be assessed directly in vivo in humans. It can only be estimated from forward dynamics simulations or inverse dynamics techniques by using kinematic, kinetic and EMG measurements in combination with computational modelling [42, 43].

Alternatively, the measured external contact force or moment caused by muscle actions can be used as proxy variable for internal muscle force. External forces and moments can be seen as a function of a patient’s neural drive (i.e. the ensemble of action potentials of all active motor neurons). At present, EMG represents the only noninvasive methodology for interfacing indirectly with the nervous system in patients [44]. Establishing relationships between neural and mechanical variables has been addressed using model-free machine learning approaches (e.g. [45]) and model-based methodologies (e.g. [46]). The latter can be used to estimate internal neuromuscular and mechanical variables, such as muscle force (e.g. [42]), joint moment (e.g. [47]), joint compressive force (e.g. [48]), joint stiffness (e.g. [49]), joint angles (e.g. [50]) or metabolic energy conversion (e.g. [51]) that otherwise could not be viably measured experimentally.

Since conventional bipolar EMG may provide imprecise muscle activation estimates and furthermore cannot simultaneously provide estimates of activation in different directions (e.g. medio-lateral vs. longitudinal) and zones (distal vs. proximal), which is important in the context of force or moment direction, the possibility of employing high-density multichannel EMG should be considered instead ([52]).

**Fig. 1:**
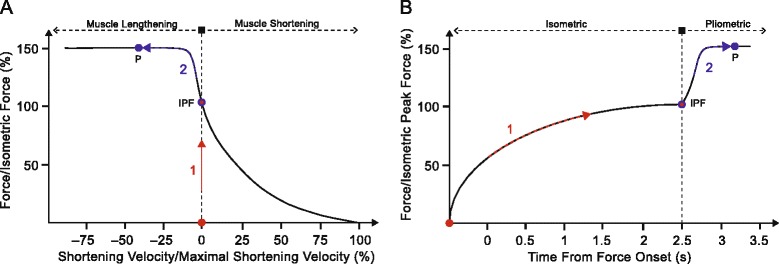
Robotic assessment of the highest force for a given maximal intended force task. The two steps during the maximal intended force exertion of the performer are denoted both in the generic length-tension relationship (**a**) and time-force relationship during the robotic assessment of peak force (**b**). Step 1: the performer reaches isometric peak force (IPF) during an all-out effort at short (30% of peak range of motion) muscle-tendon length against the rigid and fixed robot arm. Attaining isometric peak force may take up to several seconds (2.5 s in this example), depending among other things on the magnitude of the attained force. Step 2: Immediately after attaining the individual’s IPF (automatically detected by the robot), the robot smoothly increases its force in the opposite direction while the performer tries to brake the movement of the robot arm. As the robotic force increases, the performer needs to increase the lengthening velocity (Step 2, A) in order to accomodate for the increased robotic force until the point is reached, where the force is velocity-independent (force plateau, P). For safety reasons, the range of motion for the pliometric action should be strictly controlled (to 60% of peak range of motion). Isometric, maximal intended isometric muscle action against fixed-position robotic exoskeleton; Pliometric, maximal intended pliometric muscle action against the robot-imposed force increase above IPF

Because of the generic force-velocity relationship, the highest force for a given motor task should result from a maximal intended pliometric muscle action. As it takes at least 400–700 ms up to several seconds to reach peak force during a maximal intended isometric muscle action, we propose to follow a 2 step procedure to assess peak force for a given motor task (Fig. 1) [30, 31]. During the first step, the performer reaches isometric peak force (IPF) during an all-out effort at short (30% of peak active range of motion) muscle-tendon length against the rigid and fixed robot arm. Attaining isometric peak force may take up to several seconds (2.5 s in the example in Fig. 1), depending among other things on the magnitude of the attained force. The individual’s IPF is automatically detected by the robot. In the second step, the robot smoothly increases its force in the opposite direction while the performer tries to brake the movement of the robot arm. As the robotic force increases, the performer needs to increase the lengthening velocity (Fig. 1, Step 2, A) in order to accommodate for the increased robotic force until the point is reached, where the force is velocity-independent (force plateau, P). For safety reasons, the range of motion for the pliometric action should be strictly controlled (i.e., to 60% of peak active range of motion).

Another common procedure is to estimate a patient’s strength as a function of joint angle. This is achieved through sequential isometric actions over a certain range of motion (ROM) that result in an angle-moment curve. Such angle-moment curves should be used and interpreted with caution. In vivo, muscle force and moment arm both change throughout the ROM, as does neural activation. Hence, the joint angle where the moment of a force reaches its highest value is not necessarily the joint angle where either muscle force or moment arm are highest [53]. Furthermore, as the moment of a force around a joint represents the interaction between muscle and joint properties and because the exact relationship between the joint angle-muscle and length-tension functions is unknown for most muscles, the application of the generic muscle length-tension curve to the in vivo assessment of a patient’s muscle-joint system is questionable.

#### Force direction error

Force is a vector quantity in that it has both magnitude and direction. Force direction is intrinsically linked to the specific motor tasks of the activated muscles, which in turn are mediated by the selective recruitment of motor unit clusters. As a result of neuromuscular compartmentalization, a single muscle can comprise several distinct regions that each exert a different motor task. Consequently, depending on the type and severity of the neural impairment, neurological patients (e.g. after stroke) may lose independent control over single neuromuscular compartments and muscles, and produce an abnormal muscle (co-)activation pattern.

The resulting FDE describes the reduced ability to exert force in the desired direction during motor task execution. It can be defined as the angle between the force vector recorded by the sensors of the robotic device and the unit vector aligned with the theoretical direction of movement. The angle is calculated at each sampling point and averaged across the trajectory [35, 54].

Similarly, FDE can be defined by the external mechanical work. Perfect correspondence between a path direction (given by the assessment device) and the participant’s movement direction occurs when the force vector component perpendicular to the displacement is zero. This scenario minimizes the force required to follow a path and thus increases movement “smoothness”. Conversely, suboptimal directions of the actual forces may cause deviations from a given trajectory during movements. Thus, magnitude and direction of vector components gives information about movement control resulting from differential activation of neuromuscular compartments and the underlying “smoothness” of a movement.

#### Critical force and fatigue

When the time of tolerance is plotted against particular isometric maximal intended force or moment outputs, the relationship is curvilinear, with the ability to exert high force or moment falling away more sharply at higher compared to lower forces or moments. Mathematically, the force-duration relationship is described as being hyperbolic. When exercise tolerance is considered, the force-asymptote is called “critical force” (CF) [55]. Alongside with the peak force value and the curvature constant obtained during the same test, CF provides an index of critical neuromuscular fatigue threshold. The same holds true for the assessment of critical moment (CM).

**Fig. 2:**
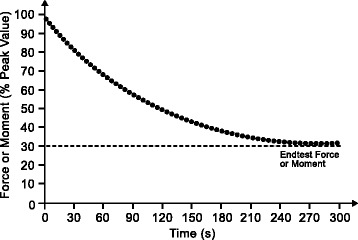
Schematic 5-min all-out test to determine critical force or critical moment. Dots represent 60 maximal intended isometric muscle actions for a given motor task with a 60% duty cycle (3 s force exertion, 2 s rest). The achieved end-test force or end-test moment corresponds to the critical force and critical moment, respectively, which are indicative of an individual’s neuromuscular fatigue threshold

We suggest to assess CF and CM by using a 5 min all-out test following the protocol of Burnley [56]. The test consists of 60 maximal intended isometric muscle actions using a 60% duty cycle (3 s force exertion, 2 s rest, Fig. 2). Under these test conditions, the end-test moment (which corresponds to CM) occurs at approximately 30% of maximal intended isometric moment, meaning that during the test the moment falls before reaching stable values at approximately 30% of maximal intended isometric moment [55, 56].

Interactions between performance fatigability (i.e., the decline in an objective measure of performance, such as force during a CF assessment) and perceived fatigability (i.e., changes in the sensations that regulate the integrity of the performer) specifically limit physical and cognitive function and determine fatigue, and both need to be assessed in order to define the neuromuscular state and the specific psychobiological condition underlying the force assessment [57, 58]. Perceived fatigability encompasses trait and state levels of fatigue, which can be assessed through scales and questionnaires that rely on self-reported responses about the preceding several days or weeks (trait level) and the measurement of fatigue at a specific instant in time during a fatiguing task (state level). The latter can be accomplished by asking the performer to instantaneously answer one or more questions about the level of fatigue or to rate the perceived exertion and pain during the exercise test. One instrument often used to assess the state level of fatigue is the visual analog scale [59].

Summarizing, the decline of force or moment with repeated isometric muscle actions (i.e., the performance fatigability), as characterized by the shape of the curve and the end test force or moment (Fig. 2) can be used in conjunction with the perceived fatigability to characterize the fatigue characteristics of patients for both intra-subject (e.g. affected vs. non-affected limb) and inter-subject (e.g. patients vs. healthy individuals) comparisons.

#### Impulse or rate of force development

For short, high-intensity movements, the impulse-generating capability of muscle is the key determining factor of performance [60]. Consequently, impulse, or as a proxy, the rate of force (or rate of moment) development [RFD (RMD)], is a measure to characterize a patient’s or athlete’s “explosiveness” [61]. The impulse, that is the force-time integral, is calculated from the force-time curve for a specified time interval (i.e. at 50, 100, 150 ms from force onset). RFD is calculated as the slope of the linear function *F* = f(*t*) from the force measurements at specific points in time (i.e. at 50, 100, 150 ms from force onset). From these measurements, either overlapping (*I*_0–50 ms_, *I*_0–100 ms_, *I*_0–150 ms_ and RFD_0–50 ms_, RFD _0–100 ms_, RFD_0–150 ms_ for impulse and RFD, respectively) or sequential (*I*_0–50 ms_, *I*_50–100 ms_, *I*_100–150 ms_ and RFD_0–50 ms_, RFD _50–100 ms_, RFD_100–150 ms_ for impulse and RFD, respectively) calculations can be performed. For the assessment of the “explosiveness” of a patient’s movement through impulse (or RFD), we recommend to apply the practical considerations given by Maffiuletti et al. [61].

We advise against using acceleration period data of “isokinetic” miometric actions and derived parameters such as torque acceleration energy (TAE) because they are affected by the different velocity control mechanisms of dynamometers used for assessment.

### What is normal

Normative data (e.g. “reference” values) are popular for classifying the patient’s muscle function relative to healthy controls, for comparing muscle function in individual patients or patient groups, to follow its natural course in health and disease, and to assess the effectiveness of therapeutic interventions. There exist normative data for isokinetic moments of upper extremity muscle groups [62–65]. However, when comparing individual measurement results to normative data, several confounding variables such as age, gender, handedness and muscle mass should be considered. Comparisons to the contralateral muscles or limb, as well as agonist to antagonist muscle groups may enable to detect imbalances in pathologies.

The National Isometric Muscle Strength (NIMS) Database Consortium established a normative database for the population of the United States in 493 adult healthy individuals by using 10 muscle groups [66]. Meldrum et al. determined median and percentile predicted peak isometric moment values stratified by age and gender for 9 muscle groups in 494 healthy individuals in Ireland [67]. Tawil et al. computed composite scores (Z scores) for the assessment of patients suffering from facioscapulohumeral dystrophy (FSHD) based on the elbow flexion moment determined in 32 healthy individuals [68]. This normative database was enlarged to 168 healthy subjects later on [69]. Hogrel et al. published a normative database for isometric peak moment values for 27 motor tasks based on a sample of 315 healthy French men and women of 20–80 years of age [70].

Several fundamental caveats need to be considered when trying to establish and apply such normative values for comparability of assessment variables between individuals. For instance, a fundamental problem is that muscle function should be normalized to the design parameters of the individual’s musculoskeletal system (Table 1), which are often not known. In practice, the measured values should at least be normalized for body mass or lean mass. Another issue is that in order to compare the measured values of muscle function with normative data, the data must have been obtained under the same experimental conditions (standardization problem). Furthermore, it should be ascertained for each motor task whether the obtained assessment variables are heteroscedastic (increasing variability with increasing values) or homoscedastic. In this context, it has been shown both in orthopedic patients and healthy individuals that repeated grip “strength” trials with submaximal and maximal effort produce homoscedastic data sets [71]. The data showed that larger mean moments did not yield larger standard deviations (SD) and that the lack of proportional change between the means and SD exists for both within-subject and between-subject grip strength scores [71]. Thus, care must be exercised when the coefficient of variation (CV, a measure for relative variability) is used to interpret results from “tracking ability” or “force steadiness” assessments. This is because weaker patients will show biased outcomes expressed as inflated CVs.

**Table 1:** Basic Musculoskeletal Design Parameters Affecting Muscle Function

Muscle Property Affected	Design Parameter
Maximum force, tension, and moment	Physiological cross sectional area (number of parallel sarcomeres) Areal distribution of muscle fiber types
Force	Pennation angle
Fatigability	Fiber type distribution (number and area)
Maximum moment	Moment arm
Velocity of excursion	Muscle fiber length (total number of serial sarcomeres) Areal distribution of muscle fiber types
Submaximum force at given shortening velocity	Muscle fiber length
Range of Motion	Muscle fiber length Tendon length
Damping, energy storage	Tendon length
Relative stiffness of a muscle-tendon unit	Tendon length/muscle fiber length
Relative muscle-joint properties	Fiber length/moment arm

Up to date, there exist no normative values for peak force obtained by upper limb robotic assessments. Also, the upper limb motor task eliciting the highest peak force (i.e. maximum force) for a given target muscle (group) is unknown. Similarly, reference values for force direction, mechanical work, critical force (or moment), fatigue, and impulse are currently missing.

## Discussion and Conclusions

### Recommendations for measurement

#### Compliance

Any compliance or deformability within the robotic system may affect joint angle and velocity and lead to force attenuation. Such effects are undesired since they may confound the measurement outcomes [72]. With respect to the effect of compliance on the joint angle, the external (robot) moment is measured around a fixed axis of rotation assuming that the tested joint axis is always in alignment with this. However, there is usually misalignment of the joint and the device axes of rotation. The misalignment originates from the compliance of the performer’s soft tissues and the device’s paddings, leading to a non-rigid connection between the performer’s body segments and the robot/dynamometer arm and seat. The non-rigid connection in turn facilitates movement of the segment relative to the robot [73]. To minimize this, it is proposed that joint and robot/dynamometer axes are aligned under active (submaximal isometric) and not passive conditions, near the primary joint angle of interest and separately for reciprocal actions (e.g. extension and flexion) and/or to correct for axis misalignment [73]. Furthermore, the components of the measurement device should be as stiff (high Young’s modulus) as possible to minimize force attenuation, but without causing discomfort or pain to the performer. Consideration of these factors should also help to reduce baseline noise amplitude and thus improve the detection of muscle action onset.

#### Force tasks vs. position tasks

The neuromuscular mechanisms responsible for the decline in force during a fatiguing isometric elbow flexor muscle action depend on the specific demands of the task being performed, such as relative force magnitude and load compliance [74, 75]. With respect to load compliance, two types of motor tasks can be distinguished: One type of task, a so-called “force task” requires the performer to exert force against a rigid force transducer under isometric conditions and to match a defined submaximal target force. The other task, a so-called “position task”, requires the performer to keep the joint at a defined angle during an isometric action while supporting an equivalent inertial load (“compliant”). Based on appropriate feedback signals, the performer is instructed to “maintain force” during the force task and to “maintain position” during the position task. However, in both cases the performer exerts the identical net external moment.

It has been shown that for target forces up to 30% of maximal intended force (“maximal voluntary contraction”, MVC) the time to task failure is briefer for a position task than for a force task. Conversely, endurance time for a force task is typically up to twice as long as for the position task at the same target forces [74, 75]. These results indicate that the capacity to perform a fatiguing isometric muscle action with a compliant load that is ≤30% of maximal intended force is less than that when exerting a comparable net external moment against a rigid restraint.

#### Instruction and feedback

Force measurements depend on the instructions given to the performers prior to force exertion [76, 77]. If the assessment aims at achieving the highest possible impulse (i.e. the highest possible RFD), then the performer should be instructed to exert force “as fast and hard” as possible with an emphasis on the explosiveness of the rising phase of the force curve. If the goal is to achieve peak force or peak moment the verbal instruction should be to exert force “as hard as possible”. Thus, the measurement of maximal intended force should be separated from the measurement of impulse whenever this is possible.

Similarly, the limit of task tolerance (time to task failure) may depend on the feedback and motivational cues given during the task. Thus, feedback and motivation should be standardized, too. Besides affecting the measurement value, accurate and standardized instructions are equally important to achieve a good retest reliability.

#### Normalization

Body size and body composition are factors well known to exert a substantial influence on absolute force and moment. Hence, for comparing individuals or groups of differing body size the measured values should be at least normalized. Different normalization approaches exist, albeit the allometric formula *F*_n_ = *F*/(*m* ^b^/kg) with *F*_n_ = normalized force or moment, *F* = measured force or moment, *m* = body mass or fat-free mass, and b = allometric scaling exponent, is recommended to obtain a normalized index for force and moment [78]. It has been suggested that for relatively homogenous healthy lean populations, allometric scaling exponents of 0.66 and 1.0 for force and moment, respectively, are appropriate [79]. For more adipose populations lower body mass exponents appear more suitable (0.45 and 0.68 for force and moment, respectively) [79]. Nevertheless, Folland et al. recommend fat-free mass as index for scaling force and moment to body size, and higher allometric scaling exponents are advocated in this case (0.76 and 1.12 for force and moment, respectively) [79].

#### Reliability

Retest reliability refers to the reproducibility of values of a test, assay or other measurement when the measurement is repeated in trials on the same individuals [80]. Hence, reliability concerns the monitoring of individuals for real changes, the estimation of sample size for experimental studies and the assessment of competing brands of equipment or varying technologies. Therefore, it is important to characterize the reliability of assessment measures in a standardized way, for instance by reporting the observed values and confidence limits of the typical error and changes in the mean [80]. Furthermore, to be reproducible and comparable between centers worldwide, the described assessment parameters need to be reported explicitly. These parameters include, for instance, familiarization, joint axes alignment methods (positioning and fixation), correction techniques, instruction and feedback, detection method of muscle action onset, sampling of the force signal, filtering, and calculation of results (averaging etc.).

#### “Pre-tension” and antagonist force (countermovement)

Active muscle force (“pre-tension”) prior to the onset of peak force or RFD (impulse) assessments can influence the attained peak force and peak RFD, respectively. Whereas the magnitude of peak moment is increased with pre-tensions up to 40% peak torque in elderly men and women, peak RFD is decreased when active force is present prior to the onset of an impulsive action [81, 82]. Similarly, it appears that the magnitude, duration and speed of force exertion by the antagonist muscle(s) immediately prior to the onset of the agonist(s) action may influence both RFD and peak moment [83, 84].

Therefore, it is advisable to standardize pre-tension conditions across trials, individuals and sessions to obtain reliable measures of peak force and RFD. One possible solution may be to show the baseline force on a high-resolution scale in real time during the assessment and to provide feedback to the performer in order to guarantee a stable baseline force.

## Conclusion

Robotic devices allow for sensible and reliable quantification of motor function. We give an overview of the existing assessment approaches of arm force with robotic devices and provide possible physiological endpoints for measurements. Furthermore, we propose a battery of meaningful robotic assessments for the upper extremity that take advantage of the control and sensor abilities of robotic devices. The suggested parameters encompass force magnitude (peak and maximum), force direction, moment of a force, impulse, and critical force. We believe that these parameters provide detailed insights into arm motor function of both healthy and patients and should be included into robotic assessments of the arm. They may give new insights into the course of diseases and broaden our understanding of pathologies.
